# It is not waste if it is therapy: cellular, secretory and functional properties of reamer–irrigator–aspirator (RIA)-derived autologous bone grafts

**DOI:** 10.1186/s10195-025-00835-0

**Published:** 2025-03-26

**Authors:** S. Häusner, A. Kolb, K. Übelmesser, S. Hölscher-Doht, M. C. Jordan, A. Jauković, F. Berberich-Siebelt, D. V. Spasovski, J. Groll, T. Blunk, M. Herrmann

**Affiliations:** 1 Musculoskeletal Cell Biology Group, Institute of Functional Materials and Biofabrication (IFB), University of Würzburg, Röntgenring 11, 97070 Würzburg, Germany; 2 Bernhard-Heine-Center for Locomotion Research, Chair of Orthopedics, University of Würzburg, Brettreichstr. 11, 97074 Würzburg, Germany; 3 Department of Trauma-, Hand-, Plastic- and Reconstructive Surgery (Surgery II), University Hospital Würzburg, Oberdürrbacher Straße 6, 97080 Würzburg, Germany; 4 Center for Orthopaedics, Trauma Surgery and Rehabilitation Medicine, University Medicine Greifswald, Fleischmannstraße 8, 17475 Greifswald, Germany; 5 Group for Hematology and Stem Cells, Institute for Medical Research, University of Belgrade, Dr Subotića 4, P.O.B. 102, 11129 Belgrade, Serbia; 6 Institute of Pathology, University of Würzburg, Josef-Schneider-Str. 2, 97080 Würzburg, Germany; 7 Institute for Orthopedic Surgery (Banjica), University of Belgrade, Milhaila Avramovica 28, Belgrade, Serbia; 8 Department for Functional Materials in Medicine and Dentistry (FMZ), University of Würzburg, Pleicherwall 2, 97070 Würzburg, Germany

**Keywords:** BM-MNCs, ABG, Bone grafting, Fracture non-union, Reamer–irrigator–aspirator, Bone healing

## Abstract

**Background:**

Large bone defects resulting from trauma, disease, or resection often exceed the intrinsic capacity of bones to heal. The current gold standard addressing these defects is autologous bone grafting (ABG). Procedures such as reamer–irrigator–aspirator (RIA) and conventional bone grafting from the iliac crest are widely recognized as highly effective interventions for critical-size bone defects. The early phase of fracture healing is particularly crucial, as it can determine whether a complete bony union occurs, or if delayed healing or non-unions develop. The initial composition of the bone marrow (BM)-rich ABG transplant, with its unique cellular (e.g., leukocytes, monocytes, and granulocytes) and acellular (e.g., growth factors and extracellular proteins) components, plays a key role in this process. However, despite many successful case reports, the role of ABG cells, growth factors, and their precise contributions to bone healing remain largely elusive.

**Materials and methods:**

We characterized the native cellularity of both solid and liquid RIA-derived ABG by analyzing primary, minimally manipulated populations of monocytes, macrophages, and T cells, as well as hematopoietic, endothelial, and mesenchymal progenitor cells by flow cytometry. Growth factor and cytokine contents were assessed through antibody arrays. Possible functional and immunomodulatory properties of RIA liquid were evaluated in functional in vitro assays.

**Results:**

Growth factor and protein arrays revealed a plethora of soluble factors that can be linked to specific immunomodulatory and angiogenic properties, which were evaluated for their potency using functional in vitro assays. We could demonstrate a strong M2-macrophage phenotype inducing the effect of RIA liquid on macrophages. Additionally, we observed an increase in anti-inflammatory T cell subsets generated from peripheral blood mononuclear cells and BM mononuclear cells upon stimulation with RIA liquid . Finally, in vitro endothelial tube formation assays revealed highly significant angiogenic properties of RIA liquid, even at further dilutions.

**Conclusion:**

The cytokine and protein content of RIA liquid exhibits potent immunomodulatory and angiogenic properties. These findings suggest significant therapeutic potential for RIA liquid in modulating immune responses and promoting angiogenesis. Anti-inflammatory and angiogenic properties demonstrated in this study might also help to further define and understand its particular mode of action while also providing explanations to the excellent bone-healing properties of ABG in general.

*Level of evidence*: Case-series (Level 4).

**Supplementary Information:**

The online version contains supplementary material available at 10.1186/s10195-025-00835-0.

## Introduction

The therapy of large bone defects remains challenging, especially when the bones innate capacity for regeneration is surpassed by defect size. In these so-called critical-size bone defects, surgical intervention is needed to stabilize the defect and to achieve bony union. The Food and Drug Administration (FDA) defines fractures that have not consolidated by 9 months since injury and show no signs of healing for 3 months as non-union [1].

The availability of ABG from the iliac crest is limited and associated with numerous complications. Therefore, the reamer–irrigator–aspirator (RIA) was developed. This advanced drilling device provides continuous irrigation and suction during the reaming of long bones. Irrigation fluid, marrow contents, and morseled bone fragments are aspirated and passed through a coarse filter, which captures solid bone fragments for transplantation before transferring the wash through into a closed suction bag [2]. The fluid in this phase, also referred to as “liquid waste,” is a mere byproduct of irrigation with physiological saline, used to lower intrafemoral pressure and thermal damage [3, 4]. Nevertheless, previous studies have demonstrated high levels of potent viable cells and growth factors in the RIA liquid phase [5–9]. Yet other studies have focused exclusively on mesenchymal stromal cells (MSCs) in RIA liquid and could prove high osteogenic capacity [6]. In this context, a therapeutic potential of RIA liquid seems promising, even if it is not utilized in current clinical standard therapies.

The exact composition of the transplanted BM-rich autografts, as well as its imminent impact on the local fracture microenvironment and subsequent bone healing, remains unknown. In addition to osteoconductive, osteoinductive, and osteogenic properties of transplanted bone tissue, BM mononuclear cells (BM-MNCs) are critical for the functional properties of ABG. In human BM neutrophils are the dominant cells, along with fractions of macrophages, monocytes, T cells, and other plasma cells, among others [10]. Nevertheless, the fate of these cell types once transplanted into the bone defect site is currently unknown [11]. Bone healing may be driven by surviving cells and cytokines released by both living and dying cells in the ABG. The “dying stem cell hypothesis” [12] suggests that angiogenesis, immunomodulation, and bone formation are linked to cytokines released from dying BM-MNCs. Literature highlights the critical roles of lymphocytes and macrophages in the early stages of fracture healing and bone homeostasis [11, 13]. BM-MNCs play key roles in bone injury through various direct and indirect mechanisms [13]. Immune cells, especially T and B cells, co-localize with bone cells, guiding bone matrix remodeling [14]. The CD4/CD8 T cell ratio shows that higher CD8 ^+^ levels relative to CD4^+^ T cells are linked to delayed or poor healing [15, 16]. Deficiencies in either CD4^+^ or CD8^+^ T cells affects macrophage polarization and reduces MSC recruitment and proliferation at the injury site [17]. Different macrophage types, including osteomacs, M1 pro-inflammatory, and M2 anti-inflammatory macrophages trigger healing through signaling molecules [18]. Additionally, macrophages influence nearby bone cells, impacting osteoimmunology and bone formation [19–22].

Despite the clear evidence emphasizing the importance of immune cells in bone healing, there are virtually no studies focusing on these cell populations in ABG and RIA-derived grafts in particular.

In this study, we aimed to assess the native cellular, secretory, and functional properties of RIA-derived ABG. A comprehensive characterization of minimally manipulated RIA-derived BM-MNCs was performed using multiparametric flow cytometry analyses. Cytokines and growth factors present in RIA-liquid-derived ABG were analyzed using ELISA and semi-quantitative cytokine arrays. The functional properties related to bone regeneration, including angiogenic and immunomodulatory properties, were evaluated through in vitro assays.

## Materials and methods

### Reamer–irrigator–aspirator (RIA) samples

Samples were obtained from five patients (Table 1) with atrophic non-union undergoing RIA surgery with informed written consent and ethical approval. No modification to the original surgery was made for the collection of RIA-solid and -liquid fractions. All RIA samples used in this study represent leftover materials that would otherwise be discarded as medical waste. Femurs were reamed to harvest solid RIA for ABG with the Reamer Irrigator Aspirator System 2 (RIA 2 System; DePuy Synthes, Johnson & Johnson) as described before [23]. For the present study, RIA liquid phase was obtained from all five patients. In three cases residual RIA solid materials also were available; otherwise, all ABG was transplanted.

**Table 1:** RIA donor and sample data

RIA donor	Sex	Age	RIA-liquid volume (ml)	RIA-liquid cell yield	RIA-liquid cell yield/ml	RIA-solid weight (g)	RIA-solid cell yield	RIA-solid cell yield/g
Donor 1	Male	49	3300.00	8.73 × 10^7^	2.65 × 10^4^	2.60	2.10 × 10^5^	8.08 × 10^4^
Donor 2	Male	34	3300.00	1.70 × 10^8^	5.15 × 10^4^	n/a	n/a	n/a
Donor 3	Female	58	3100.00	2.10 × 10^8^	6.77 × 10^4^	n/a	n/a	n/a
Donor 4	Male	47	3000.00	1.10 × 10^8^	3.67 × 10^4^	1.10	1.07 × 10^6^	9.73 × 10^5^
Donor 5	Male	24	4882.00	3.35 × 10^8^	6.86 × 10^4^	1.00	6.60 × 10^5^	6.60 × 10^5^

n/a, not available

### RIA sample preparation

**Fig. 1:**
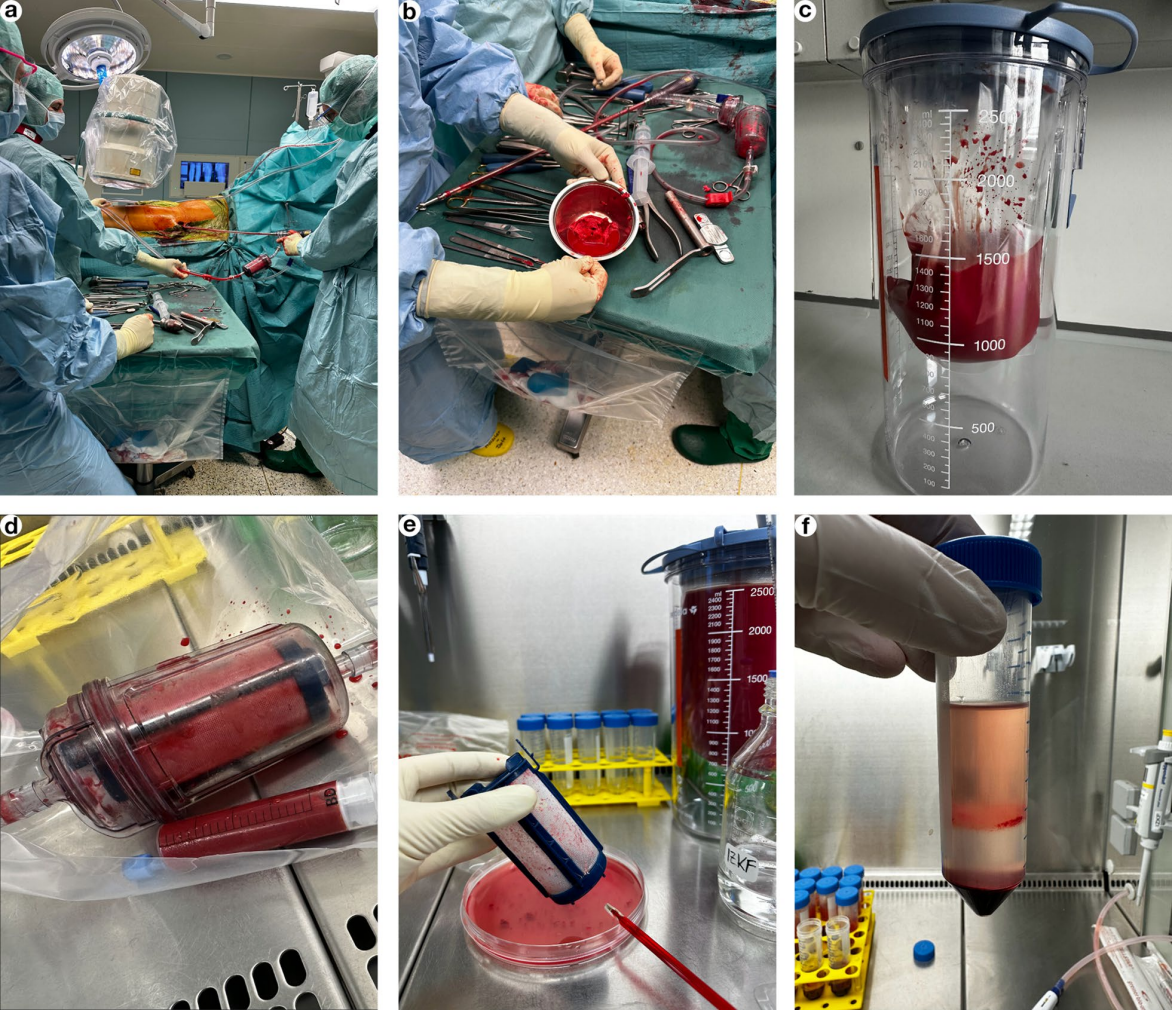
RIA sample acquisition and preparation. **a** Long bones of five patients were reamed using RIA 2 System. **b** RIA-solid BM autograft was generated in the filter unit for subsequent transplantation. **c** RIA liquid was collected in the respective fluid collection system (Medela®), and unprocessed aliquots were snap frozen for protein analyses and functional in vitro testings. **d** Residual solid RIA material was taken for isolation in the respective filtrate unit. **e** Collected solid RIA samples were scraped from the inner filter sleeve, suspended in phosphate buffered saline (PBS) and shaken until bone fragments and supernatant appeared clear. **f** After the straining and pooling of each individual solid and liquid RIA, BM-MNCs were isolated by density gradient centrifugation and analyzed by flow cytometry

RIA samples were collected directly from the operation theater (Fig. 1a–c) and processed within 6 h to ensure an unbiased, native sample constitution for analysis. All isolation steps were performed using aseptic techniques in a laminar flow hood (Methods in Supplementary Material). In brief, solid RIA material was weighed and harvested from the filter mesh (Fig. 1d, e). Volumes of RIA liquid were determined, thoroughly mixed, and aliquoted. BM-MNCs from RIA solid and RIA liquid phases were isolated by density gradient centrifugation, and remaining erythrocytes were lysed by incubation with human red blood cell lysis buffer (ThermoFisher) for 3 min. Cell pellets were washed subsequently and counted and the minimally manipulated RIA BM-MNCs directly prepared for flow cytometry. RIA liquid supernatant was 0.2 µm sterile filtered (Sarstedt) to strain all remaining debris.

### Flow cytometry

For each sample at least 1 × 10^6^ BM-MNCs were washed with PBS, centrifuged for 6 min at 1800 g, supernatant aspirated, and stained with fixable viability dye eFluor 506 (Invitrogen, #65-0866-14) for 30 min at 2–8 °C in the dark. Antibodies (Supplementary Table 1) were prepared in PBS with 1% fetal bovine serum (FBS; Capricorn) and cell pellets resuspended in surface antibody mix, vortexed, and incubated for 30 min at 2–8 °C. Cells were stained in three different panels (Ps) for monocytes and macrophages (P1: CD11b, CD14, CD80, CD163, CD169, CD204, CD206), T cells (P2: CD3, CD4, CD8, CD25, CD62L, CD127), and hematopoietic, endothelial, and stromal progenitor cells (P3: CD31, CD34, CD45LCA, CD133, CD271) unless stated otherwise. Unstained controls were incubated with PBS. Data were acquired using AttuneNxT acoustic flow cytometer (ThermoFisher). Compensation controls were performed using single-color stainings with compensation beads (Miltenyi Biotech).

Cell numbers are expressed as mean percentages of live cells. The flow cytometry results were analyzed using FlowJo™ v10.8 Software for Mac (BD Life Sciences).

### Fibroblastic colony-forming units (CFU-Fs)

RIA-liquid BM-MNCs were plated at a density of 2.5 × 10^5^ BM-MNCs in quintuplicates into 10 cm^2^ tissue culture plates (TPP) and cultured in MSC basal medium (Dulbecco’s Modified Eagle Medium [DMEM]/F-12 (1:1) GlutaMAX (ThermoFisher) + FBS + 4-(2-hydroxyethyl)-1-piperazineethanesulfonic acid (HEPES; ThermoFisher) at 37 °C with 5% CO_2_ (ThermoFisher, Heracell™ 150i). After 14 days, the media was aspirated and adherent cells washed with PBS (Merck) and then fixed with ice-cold methanol for 5 min. The methanol was aspirated, and crystal violet stain (ThermoFisher) was added to each well plate and stained for 10 min. The stain was aspirated and the wells washed twice with PBS. The cells were air-dried overnight, and colonies were counted manually using 10× magnification microscopy (Leica).

### Bicinchoninic acid (BCA) assay

After thawing, snap-frozen aliquots of RIA-liquid samples were diluted 1:200 in working buffer containing 2% sodium dodecyl sulfate (SDS) and analyzed for their protein content using standard BCA assay (ThermoFisher) according to the manufacturer’s instructions.

### Human antibody array kit

The RayBio Human Cytokine Antibody Array C2000 (RayBiotech, AAH-CYT-2000) was used to screen liquid RIA samples (*n* = 3) for the presence of 174 cytokines. Semi-quantitative arrays were prepared according to the manufacturer’s instructions, with a twofold dilution for all liquid RIA samples. Membranes were imaged using C-DiGit blot scanner (LICOR Biosciences GmbH).

Spot signal densities were quantified using ImageJ [24] and the respective Protein Analyzer plugin [25]. Data were processed with a blot-specific Microsoft® Excel-based analysis software tool provided by RayBiotech.

### Enzyme-linked immunosorbent assay (ELISA)

Levels of human BMP-2 and adiponectin in the RIA liquid were measured using commercial enzyme-linked immunosorbent assay (ELISA) kits (R&D Systems, #DY1065, #DY355-05). Snap-frozen RIA-liquid aliquots (*n* = 5) were used undiluted for BMP-2 and diluted 1:100 for adiponectin according to the manufacturer’s instructions.

### Osteogenic differentiation

BM-MSCs were isolated from BM-MNCs by plastic adherence as described before [26]. MSCs were differentiated into the osteogenic lineage, by incubation in osteoinductive medium (DMEM low glucose, ThermoFisher), 10%  FBS , β-glycerol phosphate (5 mM), dexamethasone (10 nM), and l-ascorbic-acid-2-phosphate (50 µg/ml) for 3 weeks. The medium was changed every 2 days. Deposited minerals were stained using 2% alizarin red staining solution and quantified as previously described [26].

### Endothelial tube formation assay (ETFA)

Green fluorescent protein-tagged primary human umbilical vein endothelial cells (HUVEC-GFP; Caltag Medsystems, P0027003, Addex Bio) were cultured in endothelial cell growth medium 2 (EGM-2; PromoCell, #C-22011) plus supplement mix (PromoCell, #C-39216) on cell culture flasks coated in fibronectin (0.1 mg/ml; PromoCell) at 5% CO_2_ and 37 °C, with subsequent media changes every 2 days. Cells were not used above passage 5 and passaged at 80% confluence for all experiments.

To assess the angiogenic properties of liquid RIA, HUVEC-GFP cells were cultured 16–20 h in starvation medium (EGM-2 without serum/supplements) before the experiment. ETFAs were performed by seeding 1.2 × 10^5^ cells per well onto growth-factor-reduced Matrigel (Corning, #356231; 10 mg/ml) in a 24-well plate, with three technical replicates per condition. Cells were either resuspended in starvation medium and seeded on collagen I (negative control), or on either Matrigel in starvation medium (background control), or EGM-2 with VEGF-containing supplement mix (positive control) or 10% RIA liquid. Five pictures per well were taken according to Berndt et al. [27], at 12 and 24 h using a 10× objective in phase contrast and fluorescence mode on an inverted microscope (DMi8, Leica). Images were analyzed using the ImageJ Angiogenesis Analyzer plugin. Among others, the mean mesh size, total tube length, number of junctions, number of nodes, and master segments were quantified as previously described by Carpentier et al. [28] (Supplementary Fig. 1).

### Macrophage polarization assay

Peripheral blood mononuclear cell (PBMC)-derived macrophages were pre-stimulated to generate “classically”- and “alternatively”-stimulated macrophages (Methods in Supplementary Materials). The polarizing effects of RIA liquid were tested directly on unpolarized M1/M2 macrophages (M1ø/M2ø) after day 6; M1/M2-polarized macrophages served as the control for expression levels achieved with respective recombinant stimuli.

### Carboxyfluorescein succinimidyl ester (CFSE) T cell proliferation assay

CFSE T cell proliferation assays were performed on PBMCs and BM-MNCs derived from femoral neck BM samples. PBMCs of four healthy volunteers (age 18–65 years) were isolated as described in Methods in the Supplementary Materials and directly prepared for CFSE labeling. Femoral BM samples were obtained from four patients undergoing total hip replacement surgery with informed written consent and ethical approval and were isolated by density gradient centrifugation as described before [26].

The effects of phytohemagglutinin-L (PHA-L) (ThermoFisher), PHA-L + RIA liquid, and RIA liquid on PBMC and BM-MNC proliferation were examined using CFSE. Cells (1 × 10^6^ cells/ml) were CFSE-labeled as described before [29]. Cells were seeded in 48-well cell culture plates (TPP) at a concentration of 1 × 10^6^ per well, and medium with PHA-L (positive control), PHA-L + 10% RIA liquid, or 10% RIA liquid only was added. After 120 h, cells were harvested, stained, and analyzed by flow cytometry. Cells were stained for typical T cell surface markers including CD3, CD4, CD8, CD25, CD62L, CD127, and CD45RO. Cell viability was assessed using fixable viability dye (FVD)-506.

### Statistical analysis

Where applicable, flow cytometry results were analyzed using FlowJo™ v10.8 Software for Mac (BD Life Sciences). Statistical analysis was performed with GraphPad Prism (Version 9.5.0 for macOS). Data sets were tested for normality using Shapiro–Wilk test. For data sets that did not show evidence of non-normality (alpha = 0.05) (one-way analysis of variance [ANOVA] followed by Šidák’s multiple comparison test) or data sets showing evidence of non-normality (Kruskal–Wallis and Dunn’s multiple comparison or unpaired *t*-test), the applied test is stated within the respective figure legends. All flow cytometry data sets represent mean percentages of live cells; all other data sets are displayed as mean values with standard deviation (SD). Differences with *p* ≤ 0.05 were considered to be statistically significant (*p* ≤ 0.05 [^*^], *p* ≤ 0.01 [^**^], *p* ≤ 0.001 [^***^], or *p* ≤ 0.0001 [^****^]).

## Results

### RIA cellular composition

The scope of our experiments was to characterize different mononuclear cell types contained in RIA solid and liquid to decipher the regenerative mechanism in ABG. We isolated RIA liquid (*n* = 5) and donor-matched RIA solid (*n* = 3) samples within 6 h from the initial surgery. Global RIA solid and -liquid sample cellularity displayed comparable levels in the investigated cell surface marker combinations. (Supplementary Tables 2 and 3).

**Fig. 2:**
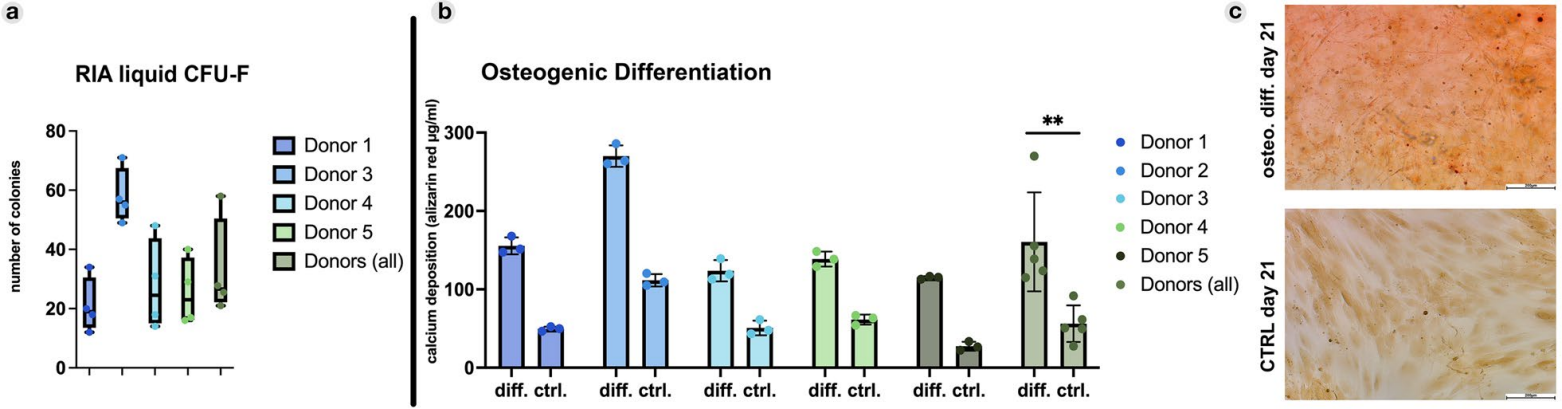
RIA-liquid-derived BM-MSC CFU-F and osteogenic properties. **a** CFU-F frequency of BM-MNCs isolated from RIA liquid. **b** Evaluation of RIA-liquid-derived MSC osteogenic differentiation potential through incorporated staining quantified by alizarin red absorption measurement. **c** Representative microscopic images of MSCs at 21 days of culture with either osteogenic or control medium. Calcium deposition visualized using alizarin red staining. Values represent the mean ± standard deviations (SD). Unpaired *t*-test was performed to assess significant differences between mean values of all osteogenic differentiations compared with control samples. Scale bar: 200 μm. **p ≤ 0.01

As a main objective in many regenerative therapy approaches, MSCs make up only 0.01–0.001% of all the cells in the BM [30]. Here, we could demonstrate that native CD45^−^ CD34^−^ CD271^+^ MSC populations occur in higher numbers in RIA solid samples, with a mean value of 0.045 ± 0.074%, and an even higher frequency in RIA liquid samples, with 0.214 ± 0.1543%, of all living cells. We further performed CFU-F assays to assess potential MSC quantities (Fig. 2a). Mean values for plastic-adherent MSC colonies ranged from 21.00 ± 3.001 to 58.00 ± 9.309, 25.5 ± 15.327, and 27.75 ± 11.328 per 250,000 seeded BM-MNCs, accounting for a mean CFU-F capacity of 0.0084 ± 0.0067% in RIA liquid. Osteogenic differentiation revealed a significant capacity of RIA-derived MSCs for calcium depositions compared with respective control samples (Fig. 2b, c).

CD45^+^ hematopoietic cells had a mean frequency of 17.50 ± 19.20% in RIA-solid samples and 39.48 ± 19.75% in RIA-liquid samples (Table 2). The hematopoietic stem cell subset of CD45^−^ CD34^+^ cells could be observed at 0.011 ± 0.014% in RIA-solid samples and 0.032 ± 0.020% in RIA-liquid samples. CD34^+^ CD133^+^ endothelial progenitor cells also displayed a slightly higher mean frequency of 0.011 ± 0.010% in RIA-solid samples compared with 0.003 ± 0.002% in RIA-liquid samples. CD3^+^ CD4^+^ lymphocytes occurred in RIA-liquid samples at 30.08 ± 9.79% and 12.87 ± 12.89% in RIA-solid fractions. CD3^+^ CD8^+^ T-cell levels accounted for 16.71 ± 16.28% in RIA-solid samples compared with 25.79 ± 8.171% in RIA-liquid samples. The respective CD4/CD8 ratio revealed a proportion value of 0.77 ± 0.853% for RIA-solid samples and a value of 1.66 ± 1.199% for RIA-liquid samples. Myeloid cells—with CD11b comprising most cells of myeloid origin such as granulocytes, macrophages, monocytes but also small T cell subsets—occurred at a mean frequency of 22.47 ± 12.45% in RIA-solid samples and 29.50 ± 9.646% in RIA-liquid samples of all living cells. Elevated levels of CD14^+^ cells could be found in RIA liquid with a mean frequency of 2.53% ± 1.351% compared with 0.565 ± 0.469% in solid RIA. Cells carrying scavenger receptor CD163 occurred with a mean frequency of 0.346 ± 0.463% in RIA solid samples and 0.219 ± 0.294% in RIA liquid samples.

**Table 2:** RIA solid and -liquid flow cytometric analyses

Cluster of differentiation	RIA-solid samples (*n* = 3)		RIA-liquid samples (*n* = 5)	
	Mean percentage (%) ± SD		Mean percentage (%) ± SD	
MSCs				
CD45^−^ CD34^−^ CD271^+^	0.045 ± 0.074		0.214 ± 0.1543	
Haematopoietic cells				
CD45^+^	17.5 ± 19.02		39.48 ± 19.750	
CD45^−^ CD34^+^	0.0111 ± 0.0135		0.0328 ± 0.0203	
Endothelial progenitor cells				
CD34^+^ CD133^+^	0.0114 ± 0.0108		0.0033 ± 0.0028	
Lymphocytes				
CD3^+^	36.53 ± 35.67		60.78 ± 18.830	
CD3^+^ CD4^+^	12.78 ± 13.89		30.08 ± 9.799	
CD3^+^ CD8^+^	16.71 ± 16.28		25.79 ± 8.171	
CD4/CD8 ratio	0.77 ± 0.863		1.166 ± 1.199	
Myeloid cells				
CD11b^+^	22.47 ± 12.45		29.50 ± 9.646	
CD14^+^	0.565 ± 0.469		2.53 ± 1.351	
CD163^+^	0.346 ± 0.463		0.219 ± 0.294	
CD163^+^ CD169^+^	0.006 ± 0.006		0.027 ± 0.036	

### Adiponectin appears highly enriched in RIA liquid

To determine the total protein content of RIA liquid samples (*n* = 5), a BCA assay was performed. The BCA protein assay revealed a mean value of 103.63 ± 41.70 mg/ml total protein contained in RIA liquid samples.

**Fig. 3:**
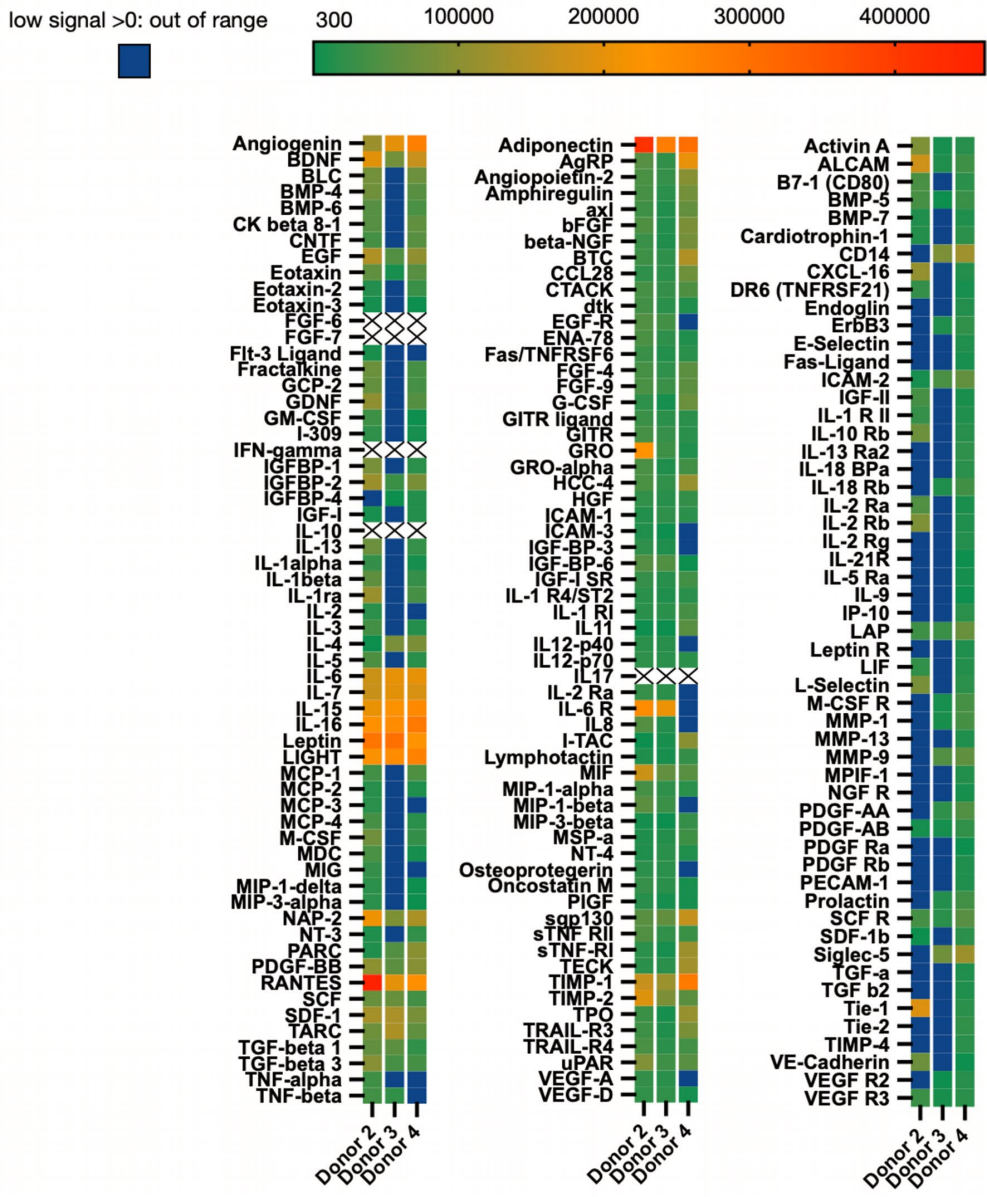
Cytokine content in RIA liquid. Antibody arrays were performed, and values were obtained using the ImageJ Protein Array Analyzer plugin. Spot signal densities are displayed in a heatmap, with the legend indicating the highest spot signal intensity levels ranging from 400,000 (red) to 300 (green). Spot signal intensities smaller than 300 but greater than zero are displayed in blue

We performed a cytokine array and used samples of the same tested donor material in the subsequent functional in vitro assays to identify contents and functional impact of soluble factors present in RIA liquid (*n* = 3). Out of 174 tested factors, 169 were detectable in at least one of the samples, while five cytokines were not detectable in any of the samples (FGF-6, FGF-7, IFN-γ, IL-10, and IL-17). Growth factors and cytokines were tested on three individual membranes (Fig. 3), where high spot signal densities were detected for angiogenin, BDNF, IL-6, IL-7, IL-15, IL-16, Leptin, LIGHT (TNFSF14), NAP-2 (CXCL-7), RANTES (CCL5), GRO a/b/g, IL-6R, MIF, TIMP-1, TIMP-2, and ALCAM. Respective adiponectin spot signal intensities were most predominant in all tested samples, across all donors.

To help interpret the semi-quantitative cytokine arrays, specific protein ELISAs of all RIA liquid samples (*n* = 5) were performed. Here especially the concentration of highly abundant adiponectin was of interest. Given the low spot level intensities observed for BMP5 and BMP7, BMP2 another prominent osteogenic factor from the BMP family was evaluated. In RIA liquid samples (*n* = 5), a mean value of 0.215 ± 0.204 µg/ml adiponectin could be detected. BMP2 revealed a mean value of 0.228 ± 0.088 ng/ml detectable in two out of five RIA liquid samples. (Supplementary Fig. 2).

### RIA liquid has angiogenic properties

**Fig. 4:**
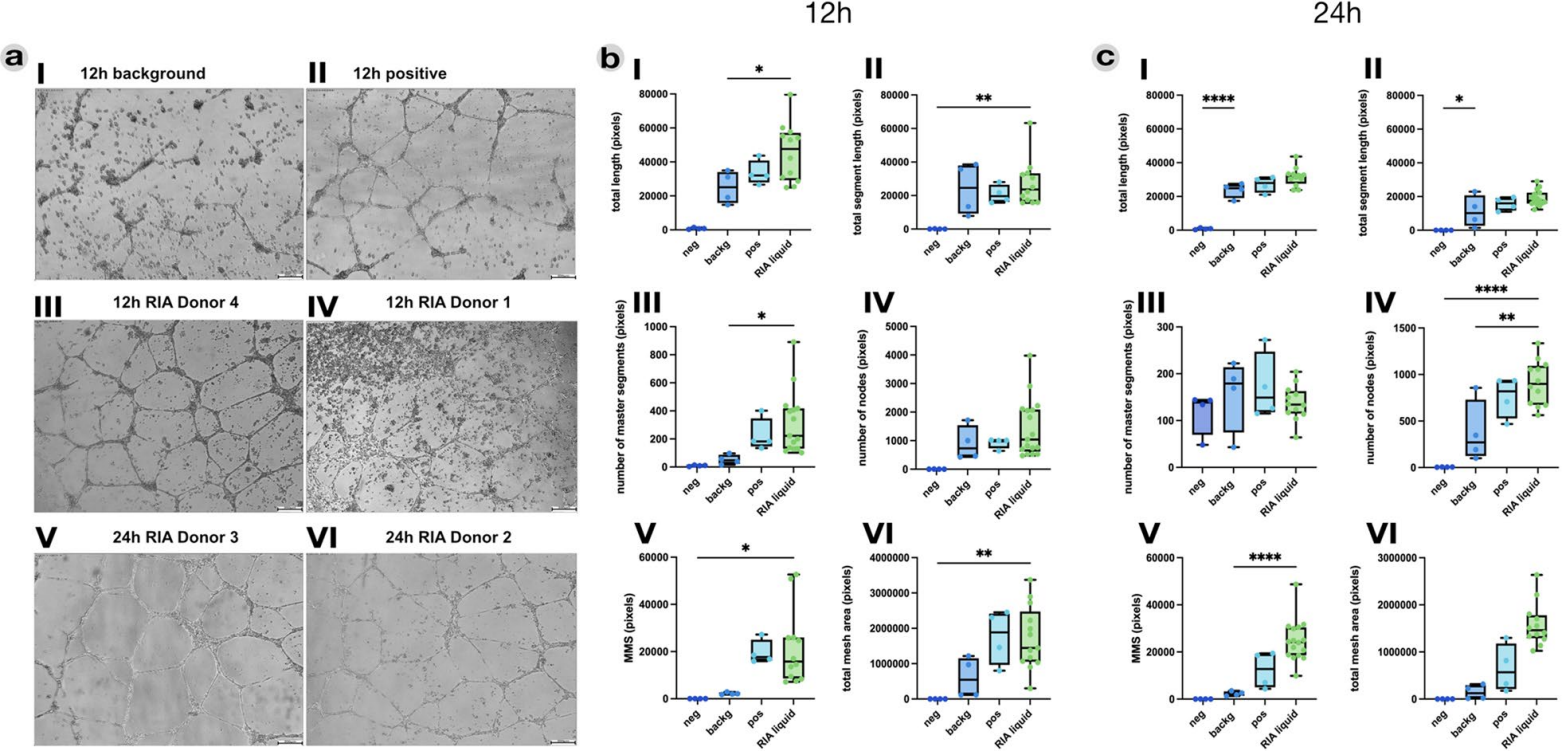
RIA liquid shows high angiogenic potential in an endothelial tube formation assay. Exemplary phase contrast images of HUVEC-seeded Matrigel after 12 h and 24 h are depicted in **a**. HUVEC were seeded in medium either without additional growth factors to account for the Matrigel itself (background control; aI), additional supplements containing VEGF (12-h positive control; aII), or supplementation with 10% RIA liquid from the respective donors at 12 h and 24 h. Mean values were taken from each of the five pictures per well taken at 12 h (**b**) and 24 h (**c**), and analyzed for total tube length (bI, cI), total segment length (bII, cII), number of master segments (bIII, cIII), number of nodes (bIV, cIV), mean mesh size (MMS; bV, cV), and total mesh area (bVI, cVI). Data are expressed as means with SD. Data were analyzed using ordinary one-way ANOVA followed by Šidák’s multiple comparison test. Scale bar: 200µm. ^*^p ≤ 0.05; ^**^p ≤ 0.01; ^***^p ≤ 0.001; ^****^p ≤ 0.0001

The capacity of endothelial cells to form tubular structures upon stimulation on Matrigel is a standard approach to test substances for angiogenic properties and was applied to test the angiogenic properties of RIA liquid. After 12 h, cells seeded on Matrigel without additional stimuli stretched across the membrane mix, creating small tubular structures, but did not build a visibly connected mesh (Fig. 4aI). The positive control resulted in a prompt progression and fusion of singular tubules toward an expanding mesh (Fig. 4aII). Donor-dependent differences were noticed for RIA-liquid-treated samples (Fig. 4aIII, aIV). After 24 h of culture, donor-specific differences were diminished since all RIA-liquid-treated samples resulted in homogeneous prominent mesh structures (Fig. 4azV, aVI). Image analysis revealed an early inductive signal of RIA liquid. RIA-treated HUVECs displayed significantly higher total length (Fig. 4bI) and number of master segments (Fig. 4bIII) compared with the background control. The number of nodes (Fig. 4bIV) was also elevated in RIA-liquid-treated samples but did not show significant differences compared with positive, background, or negative control after 12 h. The sizes of the meshes progressively increased and reached their overall maximum at 24 h, with a mean mesh size (MMS) of 24,692 pixels (±8744%) compared with a MMS of 12,399 pixels (±7758%) for the VEGF-treated positive control (Fig. 4cV), and this was highly significant in comparison to the background control, with a MMS of 2287 pixels (±976.2%). Interestingly, RIA-treated HUVEC samples could maintain their MMS and total mesh area over the course of 24 h, while contribution of big meshes decreased and tubular structures disrupted in background control and positive control samples (Fig. 4cVI).

### RIA liquid induces M2-like macrophages

To investigate the possible modes of action of ABG, and particularly the contained soluble factors with regards to immune regulatory effects on macrophages, monocytes were pre-stimulated to generate M1 (polarized), unpolarized M1 (M1ø), M2 (polarized), and unpolarized M2 (M2ø) macrophages. After pre-polarization to M1ø and M2ø, the cells were cultured with 10% RIA liquid, and their phenotypic CD profiles analyzed according to the typical surface marker expression of M1 versus M2 cells. In live/dead staining of some lipopolysaccharide (LPS)-treated M1-polarized macrophages revealed high levels of dead cells; these samples were excluded from further analyses.

**Fig. 5:**
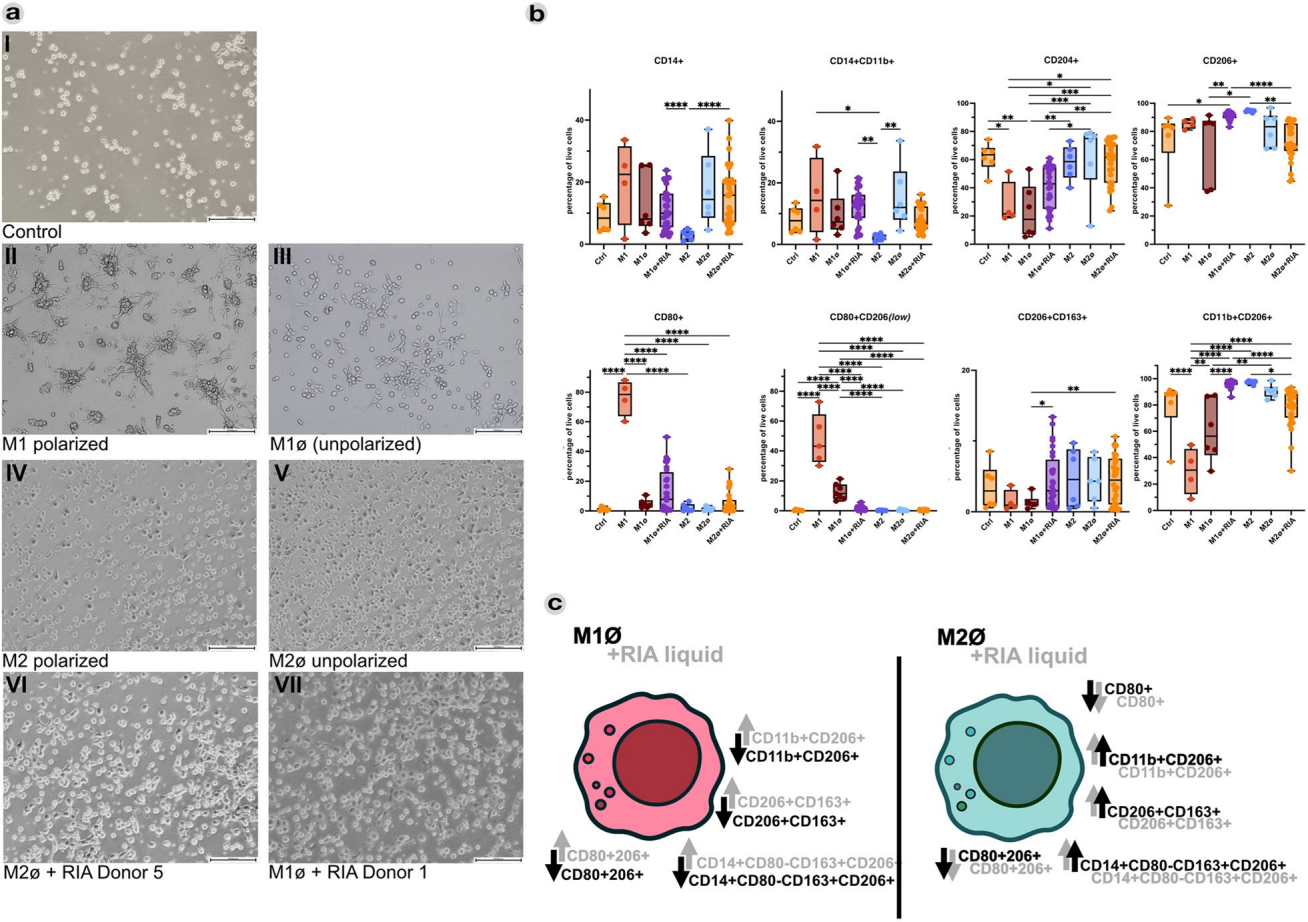
Macrophage polarization assay revealing the M2-like inducing properties of RIA fluid on pre-differentiated M1ø/M2ø macrophages. **a** Exemplary phase contrast images of CD14^+^ pre-selected PBMCs after 7 days of culture. The phenotypes of unstimulated CD14^+^ monocytes (aI), M1-polarized macrophages (aII), M1-unpolarized macrophages (aIII), M2-polarized macrophages (aIV), M2-unpolarized macrophages (aV), M2-unpolarized and Donor 5-RIA-liquid-stimulated macrophages (aVI), M1-unpolarized, and Donor 1-RIA-liquid-stimulated macrophages (aVII) differed according to the respective pre-treatment. Mean values of live cell percentages (*n* = 6) of single surface antibody and specific antibody combinations are depicted in **b**. A visual representation of the measured polarizing effects of RIA liquid on pre-stimulated unpolarized M1/M2 macrophages is displayed in **c**. For analysis, Kruskal–Wallis followed by Dunn’s multiple comparison test was performed. Scale bar: 200 µm. ^*^p ≤ 0.05; ^**^p ≤ 0.01; ^***^p ≤ 0.001; ^****^p ≤ 0.0001

Phase contrast imaging (Fig. 5aI-V) showed small, round cells in controls, with enlarged elongated M1 and rounder M2-like phenotypes, confirming successful generation of classically as well as alternatively activated macrophages. M1ø RIA-liquid-treated (M1ø + RIA) and M2ø RIA-liquid-treated (M2ø + RIA) samples resembled a mixture of both enlarged as well as elongated and roundish cells. We observed a strong decrease of CD14 and CD14^+^ CD11b^+^ cells in M2-polarized macrophages, which was not apparent in RIA-treated cells (Fig. 5b). A significant reduction of the M2-like marker CD204 compared with the control group could be determined for M1-polarized and M1ø cells, which could partially be reversed by RIA treatment. Levels of CD206, expressed preferably on M2-like macrophages, showed significantly elevated levels on M1-polarized RIA-treated cells compared with the respective M1ø control. Significantly lower levels were detected for M2ø + RIA-derived macrophages compared with the respective M2-polarized control. Typical M1-like surface markers, such as CD80 and CD80^+^ CD206(low), exhibited significantly higher expression levels in M1 cells, compared with their M1ø counterpart. This elevated expression was not observed in any of the RIA-treated samples. Typical M2-anti-inflammatory-like CD206^+^ CD163^+^ cells were significantly elevated in both M1ø + RIA and M2ø + RIA macrophages in direct comparison to M1ø macrophages. Levels of CD11b^+^ CD206^+^ M2-like macrophages were lowest in M1-polarized macrophages and elevated in M1ø + RIA cells compared with M1ø cells. In M2ø + RIA samples a slight reduction of CD11b^+^ CD206^+^ levels compared with the M2-polarized macrophages could be determined. Figure 5c illustrates significant changes in surface marker levels between unpolarized and RIA-polarized macrophages, highlighting RIA liquid's ability to promote M2 polarization in both M2- and M1-pre-polarized macrophages.

### RIA liquid supports CD4^+^ CD25^+^ and especially regulatory T cells (Treg)

**Fig. 6:**
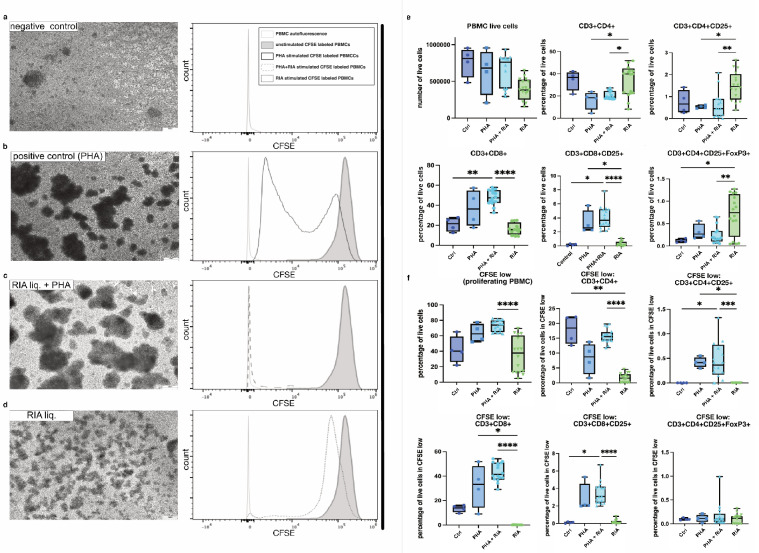
Proliferation analysis of CFSE-labeled PBMCs after stimulus. In **a**–**d** exemplary phase contrast images of PBMC cultures, adjacent to representative examples of CFSE fluorescence profiles of unstimulated (negative control), PHA-stimulated (positive control), PHA + RIA-liquid-treated, and RIA-liquid-only-stimulated samples are depicted. The grey open histograms show the autofluorescence of control samples not labeled with CFSE. The grey solid histograms show CFSE positive signals. The black open histograms show PHA-stimulated CFSE-labeled PBMCs. The grey dashed histogram shows the CFSE-labeled PHA + RIA-liquid-stimulated PBMC profile. The grey dotted histogram shows the CFSE-labeled RIA-liquid-stimulated PBMCs profile. PBMCs were exposed to either PHA, PHA + RIA fluid, or RIA liquid, and cell proliferation was examined after 120 h of culture. In **e** and **f** the numbers of all living cells, proliferating cells, and different T cell subtypes in all living cells are depicted. Cell levels, except for total cell numbers, are expressed as mean percentages of live cells. Box plots show all underlying donor samples and whiskers the minimum and maximum values. The comparison of T cell proliferation after PHA-L alone, PHA-L + RIA liquid, or RIA liquid stimulation alone was done with Kruskal–Wallis, and Dunn’s multiple comparison test was used to compare the mean percentages of cell proliferation between simulations and controls. Scale bar: 200 µm. ^*^p ≤ 0.05; ^**^p ≤ 0.01; ^***^p ≤ 0.001; ^****^p ≤ 0.0001

PBMCs were stimulated with either PHA, PHA in combination with RIA liquid, or RIA liquid alone. PBMC cultures exhibited characteristic rosetting (leucoagglutination) upon PHA stimulation, as shown with corresponding CFSE profiles, which was not observed in untreated controls (Fig. 6a). RIA liquid induced loose rosetting with more non-rosetting cells and no large agglutinates.

Flow cytometry analyses of different T cell subsets of PBMCs revealed an increase in frequencies of CD3^+^ CD8^+^ and CD3^+^ CD8^+^ CD25^+^ cells upon PHA stimulation independently from the RIA treatment, while RIA liquid treatment alone led to significantly elevated levels of CD3^+^ CD4^+^ CD25^+^ Foxp3^+^ Tregs compared with untreated controls. Levels of CD3^+^ CD4^+^ and CD3^+^ CD4^+^ CD25^+^ T helper cells were significantly higher upon RIA liquid treatment alone compared with both PHA-treated groups (Fig. 6e). A trend of increased levels of proliferating cells, visible in reduced CFSE intensity, were observed in both PHA-treated groups, while RIA liquid alone did not affect cell proliferation (Fig. 6f). In particular the proportion of proliferating cytotoxic CD3^+^ CD8^+^ and CD3^+^ CD8^+^ CD25^+^ cells as well as T helper cells (CD3^+^ CD4^+^ and CD3^+^ CD4^+^ CD25^+^) were significantly lower in samples treated with RIA liquid only, compared with the PHA-stimulated groups. Of note, RIA liquid treatment did not change the cellular response to PHA (Fig. 6e, f). Taken together, while PHA polyclonal stimulation favored CD8^+^ T cell expansion, RIA liquid supported activated CD4^+^ T and Treg cells without a major effect on their proliferation.

Since ABG transplantations primarily target BM-residing cells rather than PBMCs, femoral BM-MNCs were used in an additional CFSE proliferation assay to mimic ABG conditions. Although ABG is typically autologous, donor-matching for PBMCs or BM-MNCs to the RIA liquid was, due to technical reasons, not feasible in this experimental setup.

**Fig. 7:**
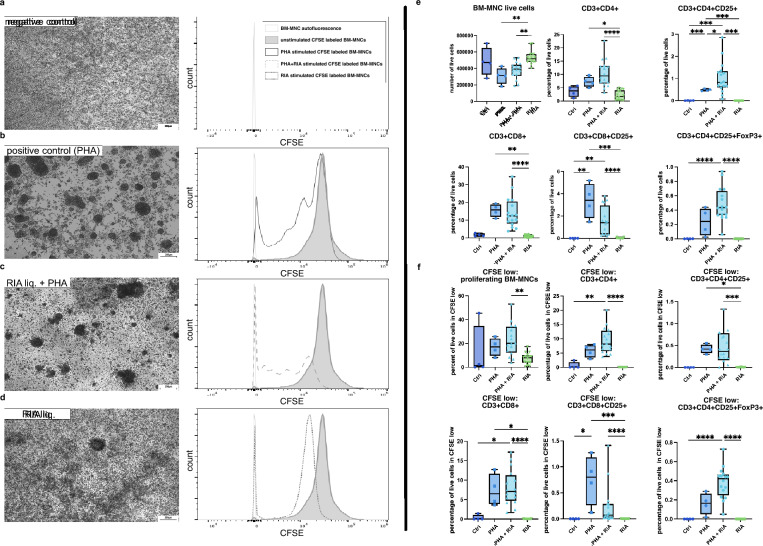
Proliferation analysis of CFSE-labeled BM-MNCs after stimulus. Exemplary phase contrast images of BM-MNC cultures, adjacent to representative examples of CFSE fluorescence profiles of unstimulated (negative control), PHA stimulated (positive control), PHA + RIA-liquid-treated, and RIA-liquid-only-stimulated samples are depicted in **a**–**d**. The grey open histograms show the autofluorescence of control samples not labeled with CFSE. The grey solid histograms show CFSE positive signals. The black open histograms show PHA-stimulated CFSE-labeled BM-MNCs. The grey dashed histogram shows the CFSE-labeled PHA + RIA-liquid-stimulated BM-MNC profile. The grey dotted histogram shows the CFSE-labeled RIA-liquid-stimulated BM-MNCs profile. In **e** and **f** numbers of all living cells, proliferating cells, and different T cell subtypes in all living cells are depicted. BM-MNCs were exposed to either PHA, PHA + RIA-liquid, or RIA liquid alone, and cell proliferation was examined after 120 h of culture. Cell levels, except for total cell numbers, are expressed as mean percentages of live cells. Box plots show all underlying donor samples (1 point = mean value of donor triplicate) and whiskers the minimum and maximum values. The comparison of T cell proliferation after PHA-L alone, PHA-L + RIA liquid, or RIA liquid stimulation alone was done with Kruskal–Wallis, and Dunn’s multiple comparison test was used to compare the mean percentages of cell proliferation between stimulations and controls. Scale bar: 200 µm. ^*^p ≤ 0.05; ^**^p ≤ 0.01; ^***^p ≤ 0.001; ^****^p ≤ 0.0001

BM-MNCs were stimulated with PHA, PHA + RIA liquid, or RIA liquid alone. No rosetting was observed in controls (Fig. 7a). PHA and PHA + RIA-liquid treatments induced rosetting resembling the PBMC CFSE assay but with smaller, denser agglutinates (Fig. 7b–d).

Flow cytometry revealed a significant decrease in the total number of live cells for the PHA- and PHA + RIA-liquid-treated group compared with samples treated with RIA liquid only. Nevertheless, both PHA-treated groups led to a significant increase in almost all T cell populations. However, only PHA + RIA-liquid treatment resulted in significantly higher levels of CD3^+^ CD4^+^ CD25^+^ Foxp3^+^ regulatory T cells in both global and proliferating (CFSE low) cells, compared with the controls and the RIA-fluid treatment alone (Fig. 7e, f). It appears as though the addition of RIA liquid to PHA-stimulated cells shifted the cellular composition to more activated CD4^+^ T cells and especially Tregs at the expense of CD8^+^ T cells. A visible trend of increased cell levels in proliferating cells and the various T cell subtypes did not reach significance between the two PHA groups.

Notably, RIA liquid alone had no significant mitogenic or immunogenic effects on BM-MNCs, unlike PBMCs.

## Discussion

The endeavor of this study was to assess cellularity, growth factor content, and possible functional properties of ABG and in particular RIA liquid, which is currently considered a waste product. No significant differences in RIA-solid and RIA-liquid cellularity could be detected; in fact, the distribution of different cell types seemed rather deterministic, as cells are washed from the reaming canal and split between the filtrate unit and liquid suction container only by means of fragment size. We could demonstrate a broad range of viable monocytes, macrophages, T cells, and endothelial cells as well as hematopoietic stem and stromal progenitor cells present in RIA liquid. Furthermore, a plethora of growth factors and cytokines relevant for bone healing could be identified. Despite its high dilution, RIA liquid demonstrated high angiogenic properties, anti-inflammatory M2-like-inducing properties in a macrophage polarization assay, and significant activating properties on regulatory T cells in peripheral blood, while in BM RIA-liquid exposure maintained the naive composition of the T lymphocyte pool without interfering with its responsiveness to PHA treatment.

The initial composition of cells within ABG plays a pivotal role. In this context, most previous studies have been focused on MSCs [6, 7, 31, 32], which have been identified in both solid and liquid RIA. It has been estimated that RIA liquid contains a mean of about 114,983 MSCs as determined by CFU-F. In some cases, this could even be an equivalent amount of MSCs contained in a full liter of undiluted BM aspirate [31, 33]. We could determine a mean number of 23,345 plastic-adherent MSCs in CFU-F assays, as well as a mean of 376,257 CD45^−^ CD34^−^ CD271^+^ MSCs in our RIA-liquid samples. The higher amount of native MSCs determined in our samples may be related to the greater amount of RIA liquid (mean 3516.00 ml) compared with the mean of 745.00 ml in the aforementioned study. These numbers also encourage observations by Cox et al. that the number of MSCs present in RIA liquid might be related to the collected volume [31]. However, MSCs identified by mere plastic adherence or the presence of specific surface markers, such as CD271, do not endorse conclusions on actual in vivo osteogenic capacity or stem cell characteristics needed for bone healing.

Moreover, increasing evidence opens the paradigm of bone healing from a rather MSC-centered approach toward an immune-factor-based process [11, 13, 34]. Thus, here we offer detailed data on the frequency of various immune cells in RIA liquid while also providing functional insights into ABG immune modulatory properties. PBMC-derived T cells showed an up-regulation in favorable phenotypes such as CD3^+^ CD4^+^ T helper or CD3^+^ CD4^+^ CD25^+^ Foxp3^+^ regulatory T cell phenotypes upon RIA-liquid stimulation while exerting only low mitogenic and immunogenic activity. CD3^+^ CD4^+^ cells are indispensable for orchestrating the immune response and have also been implicated in improved bone healing outcomes [16]. RIA liquid demonstrated further potential toward increased responsiveness of BM-MNC-derived T cells in combination with a potent mitogenic stimulus. Increased levels of CD3^+^ CD4^+^ CD25^+^ Foxp3^+^ regulatory T cells could only be detected in both PHA and RIA-liquid-stimulated samples, while RIA liquid alone did not demonstrate significant mitogenic effects. An increase of this specific T cell phenotype is favorable for normal bone healing and might also be a unique mechanism contributing to the success of ABGs.

M2-inducing properties may be linked to the rescue of healing cascades, which then might even occur in a persisting inflammatory microenvironment that is connected to delayed healing or bone non-union [18, 21, 35]. Schlundt et al. demonstrated that the transition from M1 to M2 actually aligns with vascularization events within the fracture site, implicating that M2 functions might even enable revascularization [21, 35]. An experimental upregulation of M2 macrophages at the time of injury also led to an improved healing outcome and larger bone volume [21].

Although angiogenesis is widely recognized in fracture healing, the contribution of ABG to neovascularization remains unclear. In patients with ischemia or myocardial infarcts, BM-MNCs or specific subtypes, such as CD34^+^ cells, are used to promote angiogenesis, with mixed outcomes [36]. It is to be noted that the term “BM-MNCs” is used interchangeably, and in many cases a clear distinction between BM-MNCs obtained by density gradient centrifugation or those enriched via BM aspirate concentrate (BMAC) is not made. Hence, the specific outcome cannot be directly linked to either cellular or adjacent growth factor content [37]. Interestingly Peeters et al. investigated the direct exogenous/paracrine effects of cultured BM-MNCs derived from healthy volunteers on tube formation in a HUVEC co-culture model [38]. In their study, no direct effect of BM-MNCs nor BM-MNC-conditioned media on tube formation or endothelial cell migration was observed [38], indicating that the angiogenic effects of BM-MNC therapy may be not be directly linked to living cells themselves. These results are consistent to the findings from our own ETFAs, which demonstrated significant pro-angiogenic potential of RIA liquid in absence of living cells. These results imply that short-term pro-angiogenic effects in vivo may be more closely related to the direct growth factor content of the ABG than to signal molecules actively secreted by its living cells after transplantation. Notably, VEGF, as a key angiogenic factor, was detected at low levels in our cytokine array. This is in line with the findings of Wessel et al. [9]. A plethora of other pro-angiogenic factors, molecules such as angiogenin, MIF, EGF or PDGF-BB, could be identified at high levels in RIA liquid. Interestingly, prominent anti-angiogenic factors such as tissue inhibitors of metalloproteinases (TIMPs), which have been shown to inhibit tube formation in vitro, could also be detected [39, 40]. Nevertheless, in our functional endothelial tube formation assay, RIA-liquid-treated HUVEC cultures showed a similar capacity for tubular structures when compared with the positive VEGF-containing control.

Despite low VEGF signals and the presence of both anti- and pro-angiogenic factors in RIA liquid, in vitro tube formation could occur unhindered. These suggests angiogenesis is not solely regulated by VEGF but also by synergistic effects of other angiogenic factors. It has already been demonstrated that VEGF alone fails to initiate the bone healing cascade and that pro-angiogenic factors hinder fracture healing when overexpressed [41–45]. While angiogenic factors play a key role for nutrient supply and in cell recruitment to the fracture site, there seems to be a fine equilibrium between considerable vascularization, or the lack thereof, in the formation of non-unions [43]. This equilibrium is also reflected in the RIA liquid we tested, where high signals of both angiogenic and anti-angiogenic factors could be detected.

While the more specific types and quantities of proteins within transplanted ABG or RIA-solid/liquid are yet to be determined, the greatest benefit lies in its physiological protein levels and, being species-matched rather than recombinant, its physiologically relevant protein structures. At an incorrect dosage, various bone-related growth factors, such as BMP-2 or TGF-β, have been demonstrated to stimulate tumors and cancers [46–48]. A previous study demonstrated that relevant protein levels to induce bone formation exist at a significantly lower physiological level, compared with preclinical and clinically utilized dosages [46]. Also correct dimerization and glycosylation of proteins is likewise critical for biological functions as suggested for BMP2 [46, 49, 50]. These factors may also contribute to the good clinical outcomes and define the mode-of-action of ABG, which contain signaling proteins at a non-supra-physiological level and in the correct structure [20, 46, 51]. We could demonstrate that RIA liquid, which is oftentimes mistakenly termed “waste phase,” contains substantial amounts of immune cells and growth factors with proven functional activity. Thus, despite its high dilution, RIA liquid might, in addition to the solid autologous bone graft, further enhance its regenerative potential. Reintegration of RIA liquid back into the procedure could also help to reduce the amount of ABG needed. This could be achieved by combining smaller amounts of ABG, such as RIA solid, with available synthetic bone substitute materials that could absorb RIA liquid for direct on-site application. Due to the high risk of non-unions, bone substitute materials can only be applied in combination with BM in a maximum mixture ratio of 1:3 in favor of the synthetic material [52–55]. Given its vast cytokine and protein content, a combination of ABG with RIA liquid bound to artificial bone substitute materials might help to overcome current limitations in dose recommendations of synthetic bone materials in complex grafting. Another relevant clinical application could be the re-concentration of RIA liquid with an already commercially available point of care device, such as the Arthrex™ Angel-System, which can process liquid BM samples into platelet-rich plasma gels suitable to support grafting procedures. Depending on the case-specific patient setting, different approaches for the re-concentration or either direct application could be feasible.

However, in the current national and international regulatory landscape, special care must be taken when it comes to the possible reintegration of minimally manipulated cells, as the definition and scope of a “minimal manipulation” is not standard and differs across countries. Also, the use and regulation on medical surplus/waste differs across countries when it comes to ethical and good manufacturing practice guidelines for later use as therapeutic option. To tackle this situation, the regulation on standards of quality and safety for substances of human origin (SoHO) intended for human application has been introduced within the European Union in 2024. In the USA, for example, reapplication of RIA liquid for therapeutic purposes could be affected by the 21 CFR 1271.10(a)(1) criterion of minimal manipulation and the 21 CFR 1271.10(a)(2) criterion of homologous use, depending on the route of application—with adjacent supporting substances or storage agent. However, therapeutic attempts may generally be feasible as a form of compassionate use when no other treatment option is available (which is oftentimes the case in non-union defects).

Given the vast quantity of potent growth factors, RIA liquid could also represent a feasible source for non-cell-based allogeneic treatments [33]. Here, we could already demonstrate possibly beneficial allogeneic effects of cell-free RIA liquid on angiogenesis as well as T cell and macrophage populations alike. Beyond its direct therapeutic application, RIA liquid could also serve as diagnostic tool to get insight in the patient’s current status. Given the complexity of the procedure and patient-specific settings, our data only manage to provide an initial glimpse into potential biomarkers present in RIA liquid. Future studies with larger datasets may enable the identification of predictive combinations, such as macrophages or lymphocytes beyond the clinically relevant CD4/CD8 ratio, to facilitate personalized therapy selection. Moreover, since RIA liquid is currently a main byproduct that is not yet transplanted in standard healing approaches, it is available for research without ethical concerns. Due to the simplicity of our experimental in vitro setting, more comprehensive three-dimensional (3D) culture models and preclinical in vivo model systems will be needed to further investigate and verify the relevant mechanisms demonstrated in this study.

## Supplementary Information

Supplementary Material 1.

Supplementary Material 2.

Supplementary Material 3.

Supplementary Material 4.

## Data Availability

The datasets used and/or analyzed during the current study are available from the corresponding author on reasonable request.

## Abbreviations

ABG: Autologous bone graft
BCA: Bicinchoninic acid
BM: Bone marrow
BM-MNCs: Bone marrow mononuclear cells
BM-MSC: Bone marrow mesenchymal stromal cells
CFSE: Carboxyfluorescein succinimidyl ester
CFU-F: Fibroblastic colony-forming unit
ELISA: Enzyme-linked immunosorbent assay
HUVEC: Human umbilical vein endothelial cells
M1ø: M1 unpolarized
M2ø: M2 unpolarized
MNC: Mononuclear cell
MSC: Mesenchymal stromal cell
PHA-L: Phytohemagglutinin-L
RIA: Reamer–irrigator–aspirator
SD: Standard deviation
