# Supplementary material for: It is not waste if it is therapy: cellular, secretory and functional properties of reamer–irrigator–aspirator (RIA)-derived autologous bone grafts
Source: J Orthop Traumatol. 2025 Mar 26;26:21. doi: 10.1186/s10195-025-00835-0 (PMC11947367; doi:10.1186/s10195-025-00835-0)
Supplement: Supplementary file 1 — Supplementary Material 1. [file 10195_2025_835_MOESM1_ESM.pdf]

Supplementary Methods

RIA sample preparation

RIA samples were collected directly from the operation theatre (fig. 1a).
Both solid (fig.1b) and RIA liquid samples (fig. 1c) were processed within 6 hours from operating room to isolation in order to ensure an unbiased, native sample constitution for flow cytometry and protein analysis. All isolation steps were performed using aseptic techniques and were performed in a laminar flow hood with sterile utensils. The minimally manipulated RIA BM-MNCs were subsequently assessed via flow cytometry on the same day.
Where applicable, residual solid RIA material was weighed within the disposable filter unit (fig. 1d), and harvested from the filter mesh with respective inner sleeve by scraping (Sarstedt) and rinsing with phosphate-buffered saline (PBS) tab (ThermoFisher) (fig. 1e). Morselized bone fragments along with BM was collected in falcon tubes, suspended in PBS tab and inverted briefly. Samples were filled up with PBS to a total volume of each 50ml and spun down for 5 minutes at 800g. Samples were strongly shaken and put down to sediment bone and tissue fragments.The supernatant was collected and the specimen tube refilled with PBS tab. The procedure was repeated until bony fragments and supernatant appeared colorless without presence visible suspended particles.
Afterwards the supernatants containing solid RIA BM-MNCs were processed for density gradient centrifugation, in the same procedure as liquid RIA samples
Single liquid RIA phases were transferred from suction bags (Medela) to sterile glass bottles (Schott) for exact volume determination and filtered using 40µm (Greiner) and 70µm cell strainers (Greiner) before aliquoting the whole volume (mean volume all RIA liquid samples 3.50-14.40 ml) into 50ml falcon tubes (Greiner).
All supernatants were centrifuged for 10 min. at 800g and, where applicable, appearing fatty layers skimmed off and discarded. Cell pellets were pooled for mononuclear cell isolation, aliquots of supernatants subsequently snap frozen in liquid nitrogen for protein and functional analysis. Pooled cell solutions were strained before re-suspension in PBS tab and gently layered on Histopaque (Sigma Aldrich) for BM-MNC separation by density gradient centrifugation for 25 minutes at 800g without breaks and lowest level of acceleration. The respective MNC containing intermediate layers were collected (fig. 1f) and pooled. Remaining erythrocytes were lysed by incubation with human red blood cell lysis buffer (ThermoFisher) for 3 minutes. Cell pellets were washed subsequently, counted and directly prepared for flow cytometry.
RIA liquid supernatant was 0.2µm sterile filtered (Sarstedt) to strain all remaining debris prior to all cell culture related in vitro applications.

Flow cytometry

BM-MNCs were isolated from the intermediate phase by density gradient centrifugation. For each sample analysis at least $1*10^6$ cells were washed with PBS tab for 6 minutes at 800g, supernatant aspirated and stained with fixable viability dye eFluor 506 (Invitrogen) for 30 minutes at 2-8°C in the dark. Antibodies (Supplementary Table 2) were diluted according to manufacturer’s recommendation in PBS with 1% FBS.
Cells were washed with PBS- for 6 minutes spun down at 800g, supernatant aspirated and stained with fixable viability dye eFluor 506 (Invitrogen, #65-0866-14) for 30 minutes at 2-8°C in the fridge.

Cells were stained in three different panels either for panel 1: monocytes and macrophages (P1: CD11b, CD14, CD80, CD163, CD169, CD204, CD206) T cells (P2: CD3, CD4, CD8, CD25, CD45RO, CD62L, CD127), and hematopoietic, endothelial and stromal progenitor cells (P3: CD31, CD34, CD45LCA, CD133, CD271)
Unstained controls were prepared using the same preparation with a mock treatment (PBS) along with the treated samples. Flow cytometry sample data were acquired using AttuneNxT acoustic flow cytometer (ThermoFisher, A24858) at a flow rate of 100µl/second. Compensation controls were performed using single-color staining of compensation beads (Miltenyi Biotech). Laser controls using performance beads (ThermoFisher) were performed beforehand to each flow cytometry assessment.

Macrophage Polarization Assay

Peripheral blood mononuclear cells (PBMCs) of 6 healthy volunteers (age 18-65 years) were isolated from leukocyte reduction system cones (residual material from leukapharesis, provided by Institute for Transfusion Medicine and Haemotherapy University Hospital of Würzburg) using density gradient centrifugation (Sigma). After intermediate phase collection, MNCs were washed in PBS (Sigma), remaining erythrocytes were lysed by incubation with RBC lysis buffer (ThermoFisher) for 3 minutes and washed subsequently. Human CD14+ monocytes were enriched using anti-human CD14 MicroBeads (Miltenyi) according to manufacturer’s protocol. Efficiency of the sorting procedure was assessed directly after magnetic sorting using flow cytometry with a CD14 purity yield of 87.91±3.01%.
$1x10^6$ million CD14+ enriched cells were seeded per well into respective 6 ultra-low attachment well plates (Corning). In order to test the polarizing effects of liquid RIA on „classically“ and „alternatively“ stimulated macrophages, M1 polarized macrophages (M1), M1 unpolarized macrophages (M1ø), M2 polarized macrophages (M2), M2 unpolarized macrophages (M2ø) were generated. For this, CD14+ cells were cultured for 6 days in RPMI 1640 (Gibco) containing 10% FBS with additionally 50ng/ml GM-CSF (Peprotech) for pre-stimulation of M1Ø and M1, and an additional 24 hours with either 50ng/ml GM-CSF for the generation of M1Ø or 50ng/ml INFγ (R&D Systems) and 50ng/ml LPS (Thermo) for the generation of M1.
For the generation of either M2ø and M2 macrophages, CD14+ cells were cultured for 6 days in RPMI 1640 (Gibco) containing 10% FBS with additionally 50ng/ml M-CSF (Peprotech) for pre-stimulation of M2Ø and M2 macrophages, and 24 hours additionally with either 50ng/ml GM-CSF for M2Ø or 20ng/ml IL-4 (Peprotech) and 20ng/ml IL-13 (Peprotech) for the generation of M2. These classically and alternatively polarized/unpolarized macrophages served as positive control. Respectively to test the effects of RIA-liquid on pre-stimulated M1ø and M2ø, cells were incubated for 24h with RPMI 1640 supplemented with 10% RIA-liquid. After 24h, cells were prepared as described beforehand for flow cytometric analyses. Macrophages were stained for CD11b, CD14, CD80, CD163, CD169, CD204, CD206 (all ThermoFisher, Suppl.Table 1). We defined M1 macrophages by high expression of CD14, CD80, M2 type macrophages with higher expression of CD206, comparatively higher expression levels of CD14, CD163 and CD206, according to previously described M1/M2 phenotypes (1-4). All cells were cultured at 37°C with 5% CO_2.

Alizarin red staining

Calcium composition was stained using 2% alizarin red staining solution (Sigma) at pH 4.2 with 10% diluted ammonium hydroxide (Sigma). Cells supernatant was aspirated, cell washed briefly with cold PBS (ThermoFisher) and air dryed. 24 well plates were extensively washed with deionized water and stained with 150ul of 2% alizarin red solution with gentle agitation for 15 min. at room temperature. Subsequently cells were washed again, before alizarin red was eluted using 10% cetylpyridinium chloride solution (Sigma). The absorption levels of triplicates of each well containing 100μl elutes were measured in 96 well plate in a microplate reader (Tecan) and results compared to an alizarin standard curve.

Supplementary Results

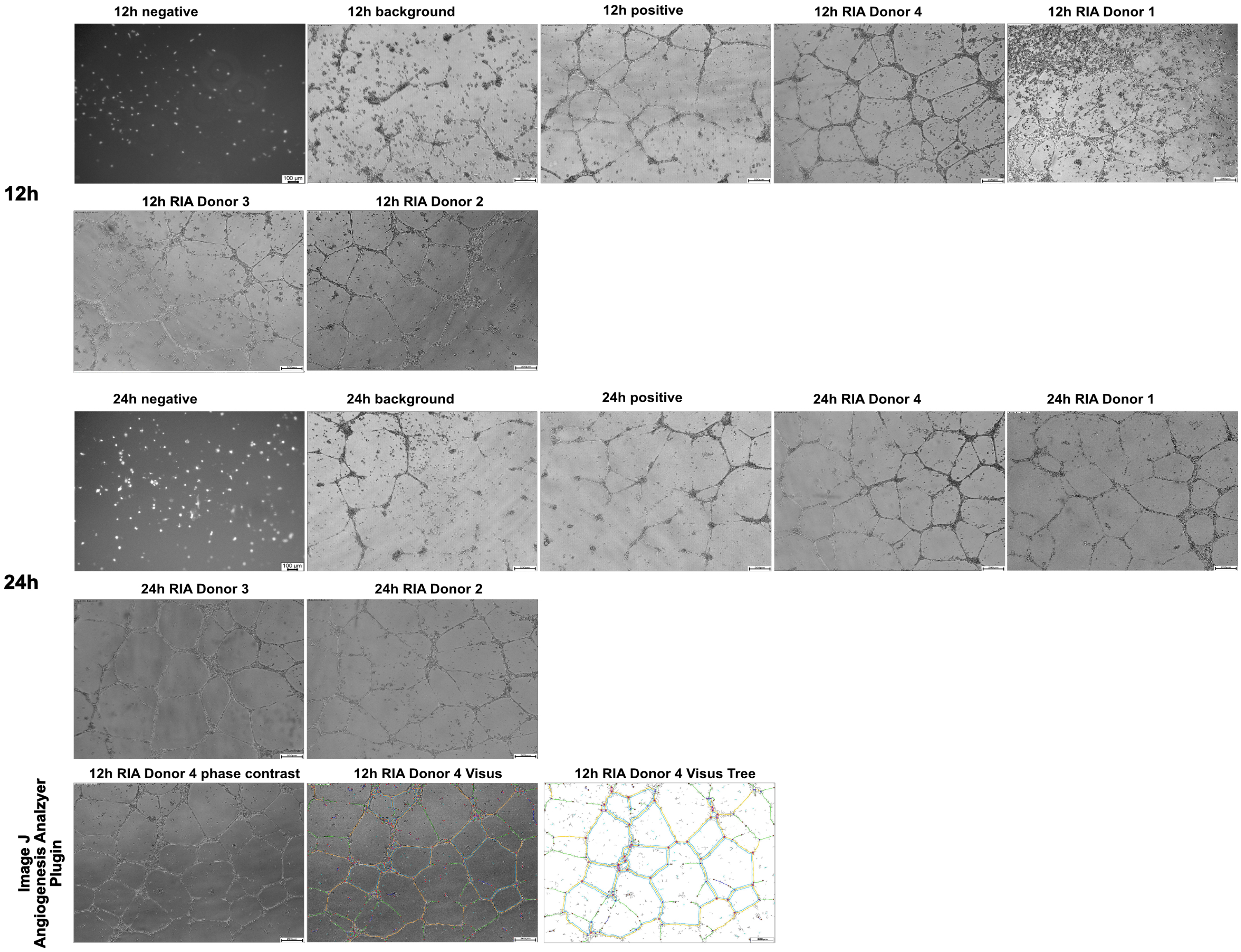

Supplementary figure 1. Endothelial Tube Formation Assay (ETFA)

Phase contrast images of HUVEC cultured in starvation medium and seeded on collagen I (kindly provided by Fraunhofer Translational Centre Würzburg) (negative control), or on Matrigel in either starvation medium (background control), or with either EGM-2 + supplement mix containing VEGF (positive control) or 10% RIA-liquid. Exemplary images also reflect on donor-specific response, like high tubular network formation in Donor 4 at 12h in comparison to Donor 1 or 3 at 12h of culture in the first and second row. Tubular network formations at 24h of culture are depicted in row three and four. Exemplary phase contrast images and respective visus tree during ImageJ image analysis using the Angiogenesis Analyzer plugin is depicted in the fifth row. Scale bar: 200µm

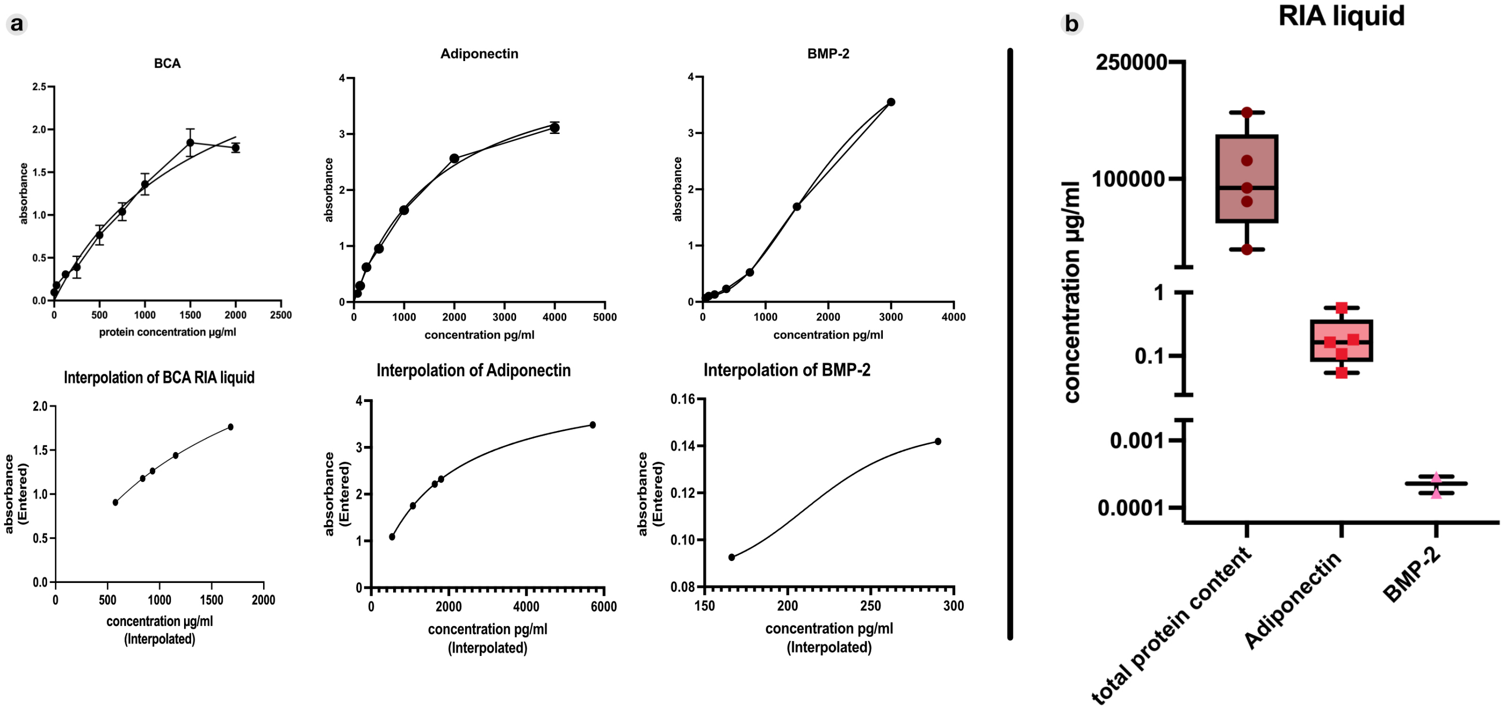

Supplementary figure 2. BCA, Adiponectin and BMP2 ELISA data set

In a) the upper respective protein standard curves for the BCA, Adiponectin and BMP-2 ELISA are depicted. Directly below, the interpolated protein concentrations as detected in RIA-liquid samples for BCA (n=5), Adiponectin (n=5) and BMP-2 (n=5) are shown. For BMP-2 only in 2 out of 5 samples protein signals could be determined.
In b) mean concentrations of either 103,628±41.696µg/ml total protein content, 0.2153±0.20415085µg/ml Adiponectin and 0.2284±0.087845ng/ml BMP-2 are depicted.

Bibliografie

1. Mantovani A, Sica A, Sozzani S, Allavena P, Vecchi A, Locati M. The chemokine system in diverse forms of macrophage activation and polarization. Trends Immunol. 2004;25(12):677-86.
2. Gundra UM, Girgis NM, Ruckerl D, Jenkins S, Ward LN, Kurtz ZD, et al. Alternatively activated macrophages derived from monocytes and tissue macrophages are phenotypically and functionally distinct. Blood. 2014;123(20):e110-22.
3. Raggi F, Pelassa S, Pierobon D, Penco F, Gattorno M, Novelli F, et al. Regulation of Human Macrophage M1-M2 Polarization Balance by Hypoxia and the Triggering Receptor Expressed on Myeloid Cells-1. Front Immunol. 2017;8:1097.
4. Heideveld E, Hampton-O'Neil LA, Cross SJ, van Alphen FPJ, van den Biggelaar M, Toye AM, et al. Glucocorticoids induce differentiation of monocytes towards macrophages that share functional and phenotypical aspects with erythroblastic island macrophages. Haematologica. 2018;103(3):395-405.
